# An unusual case of aortic metastasis from lung cancer

**DOI:** 10.15190/d.2020.3

**Published:** 2020-03-31

**Authors:** Rodica Diaconu, Roberta Florescu, Anne Cornelissen, Afify Mamdouh, Nicole Schaaps, Saskia von Stillfried, Peter Boor, Ruth Knüchel-Clarke, Ionuț Donoiu, Felix Vogt

**Affiliations:** Department of Cardiology, University of Medicine and Pharmacy of Craiova, Romania; University Hospital RWTH Aachen, Division of Cardiology, Angiology and Critical Care, Aachen, Germany; Institute of Pathology, University Hospital Aachen, Germany

**Keywords:** Autopsy, cardiovascular complications, cardiovascular disease, non-small cell lung cancer, NSCLC, hemangiosis carcinomatosa.

## Abstract

In patients with cardiovascular events, such as myocardial infarction or aortic dissection without known risk factors for cardiovascular disease, neoplastic disease should be considered as a differential diagnosis. In this report, we present a case of a 51-year old man with previously undiagnosed non-small lung cancer leading to fatal cardiovascular complications due to hemovascular spread, diagnosed post-mortem. This case illustrates the value of autopsy in unexpected deaths.

## INTRODUCTION

Primary and secondary malignancies of the aorta are very rare findings. Primary tumors involve the intimal wall of the aorta, sarcoma being the tumor of the aorta detected most often^1^. Secondary tumors involve the aorta through the adventitia, either by continuity or through secondary infiltration from lymph node metastases, and very rarely through the vasa vasorum of the aorta. There are only a few cases of secondary aortic neoplasms described in the literature^2-5^.

Secondary involvement of the aorta can occur from a variety of neoplasms. Nevertheless, the most common malignancies to present with aortic invasion are lung cancer, esophageal cancer, and thymoma^1^.

We report a case of metastatic invasion of the aortic vasa vasorum due to non-small cell lung cancer, diagnosed post-mortem.

## CASE REPORT

A 51-year-old Caucasian male, with no significant medical history or chronic ongoing treatment, was found dead in his house. In the months preceding his death, he was complaining of back pain.

At autopsy, acute myocardial infarction of the anterior wall was diagnosed as the immediate cause of death.

Multiple myocardial fibrosis areas were revealed in the anterior wall, suggesting recurrent acute ischemic events. Mild atherosclerosis plaques without significant stenosis were found in the coronary arteries. Neoplastic vascular invasion was present in the coronary vessels and lymphangiosis carcinomatosa with malignant pericardial effusion was detected.

**Figure 1:**
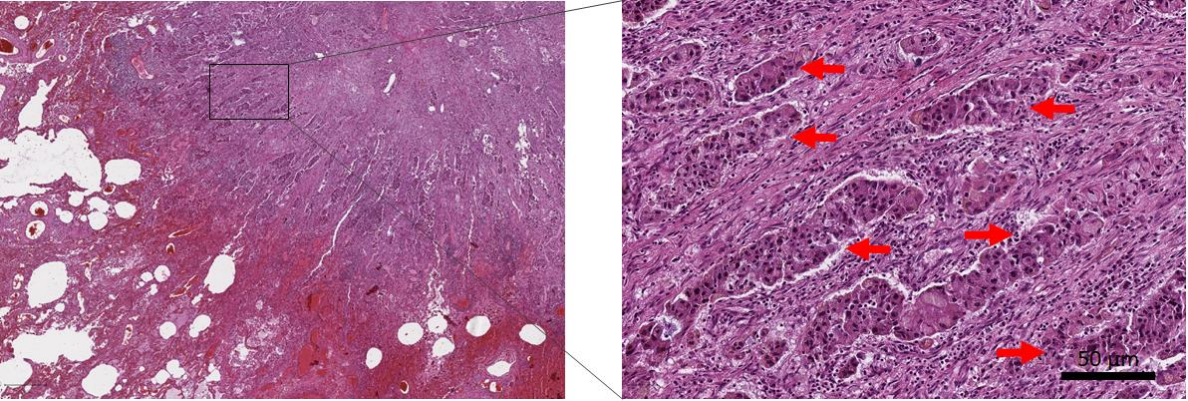
Microscopic view of tumor cells in the lung (hematoxylin and eosin staining) Red arrows point to neoplastic cells (scale bar, right panel: 50 μm).

**Figure 2:**
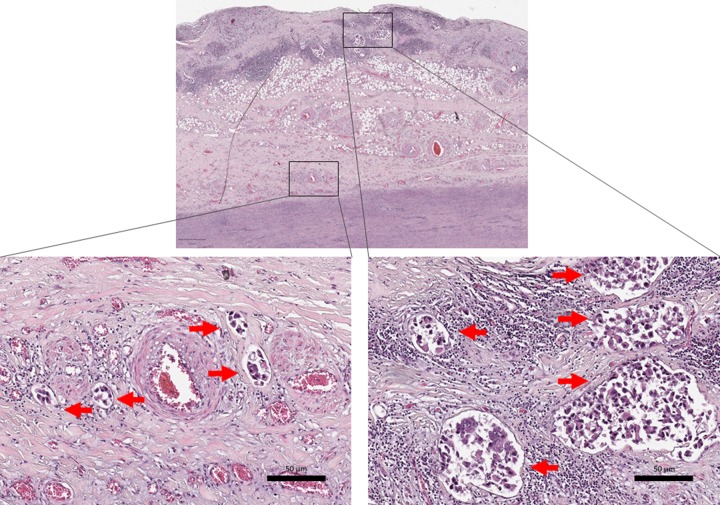
Microscopic view of tumor cells in the aorta *Left panel: *diffuse malignant infiltration of the vasa vasorum (hematoxylin and eosin staining; scale bar: 50 μm). *Right panel:* tumor cells in the aortic adventitia (hematoxylin and eosin staining; scale bar: 50 μm). Red arrows point to neoplastic cells.

Two tumors were found in the lung, the first of 1.2 cm in diameter in the left inferior lobe, and the second of 0.6 cm in diameter in the right upper lobe. Metastases were found in the intrapulmonary, hilar, jugular, parajugular and paraaortic lymph nodes and invasion was detected within the vasculature, lymphatics and perineural regions. The patient was diagnosed with non-small cell lung cancer with lymphovascular, lymphonodal and hemovascular spread T4N3M1c. The malignancy was identiﬁed to be an adenocarcinoma of the lungs (**Figure 1**). Histological examination of the aorta revealed diffuse inﬁltration of the adventitia (**Figure 2**, right panel) and hemovascular spread to the vasa vasorum (**Figure 2**, left panel).

**Figure 3:**
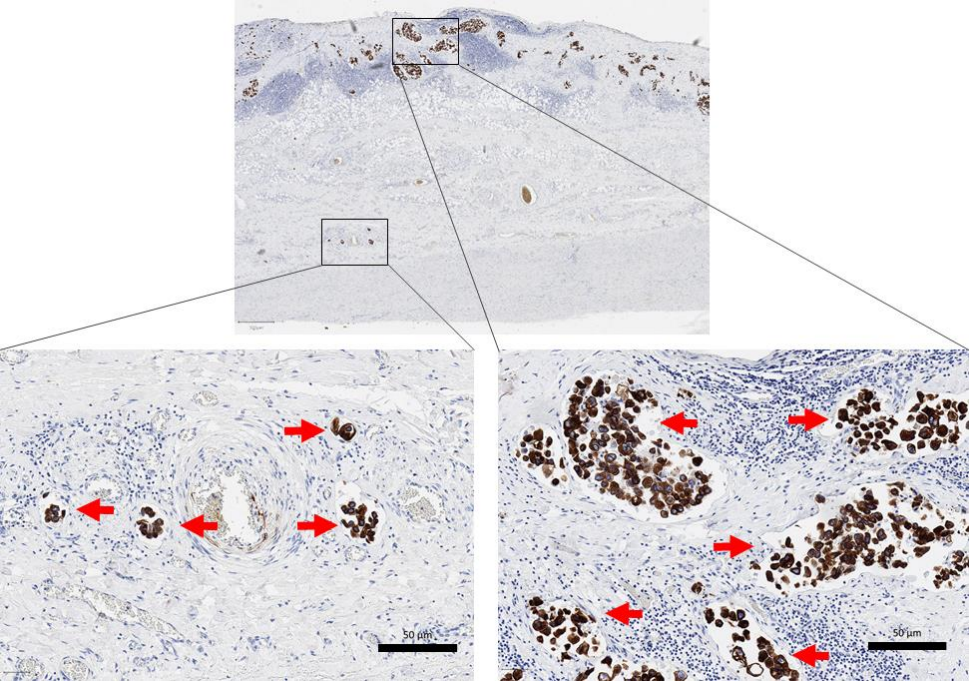
Immunohistochemistry CK7 positive Red arrows point to neoplastic cells; scale bar: 50 μm.

**Figure 4:**
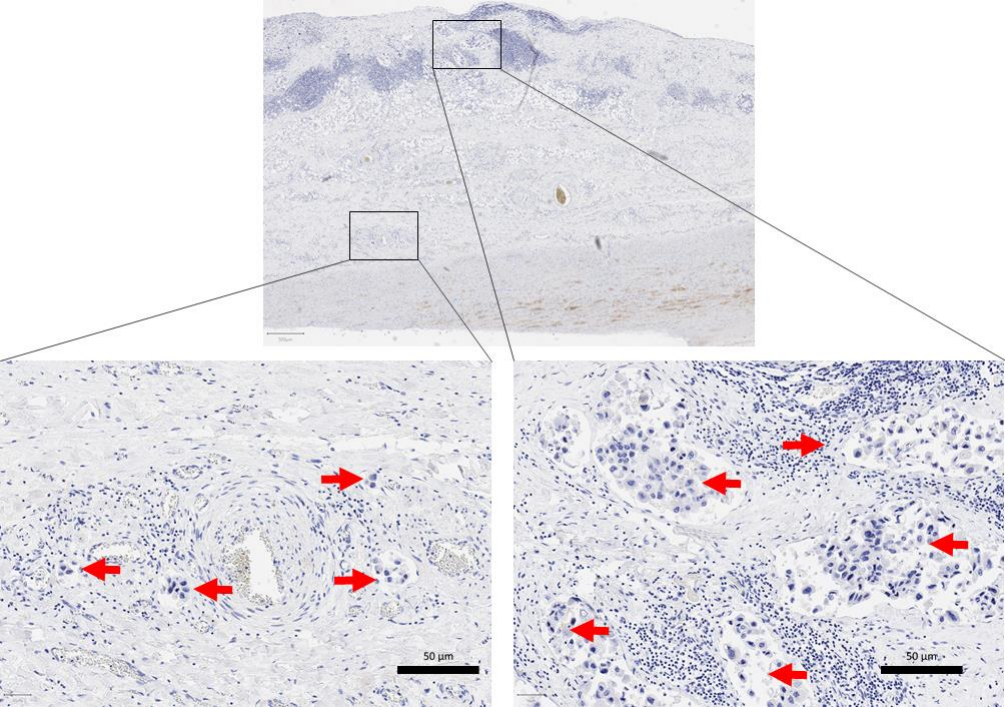
Immunohistochemistry CK20 negative Red arrows point to neoplastic cells; scale bar: 50 μm.

**Figure 5:**
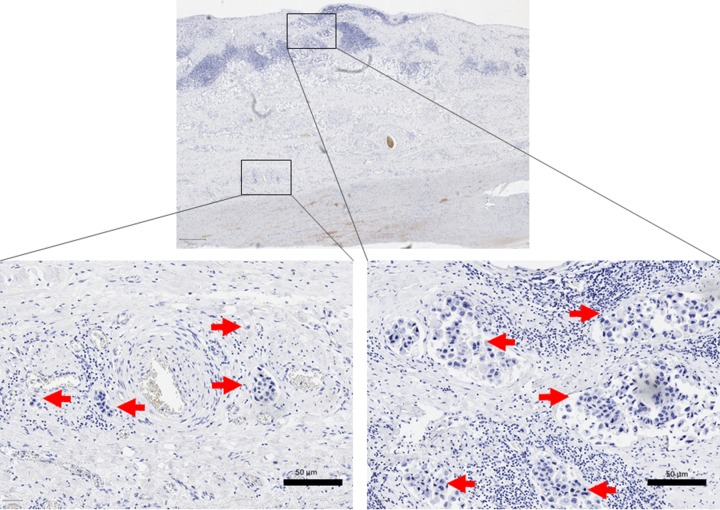
Immunohistochemistry CDX2 negative Red arrows point to neoplastic cells; scale bar: 50 μm.

**Figure 6:**
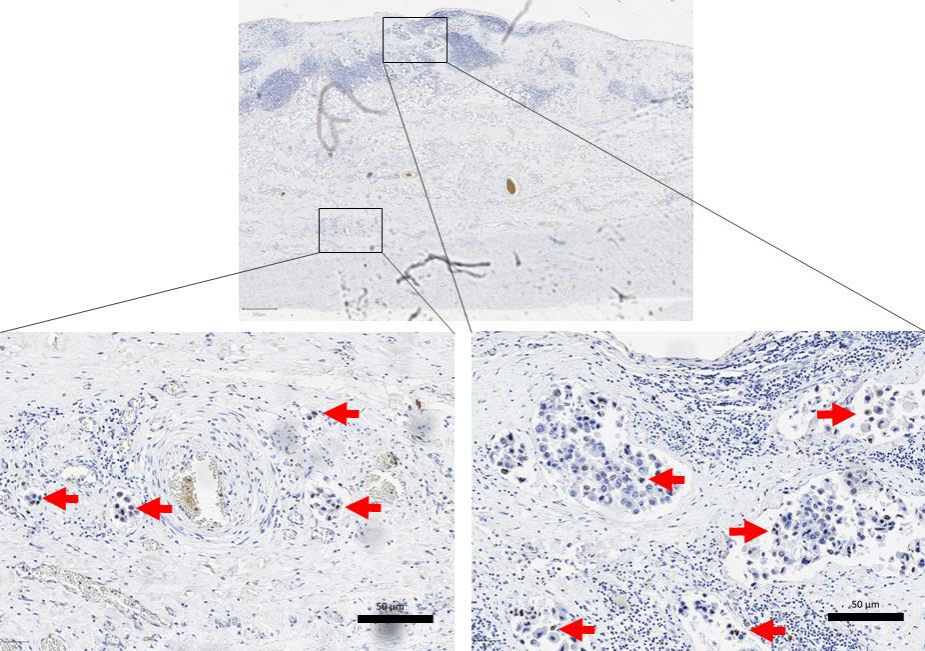
Immunohistochemistry TIF1 positive Red arrows point to neoplastic cells; scale bar: 50 μm.

Immunohistochemistry showed positive expression of cytokeratin 7 (CK7) (**Figure 3**) and negative expression of cytokeratin 20 (CK20) (**Figure 4**), typical for primary lung adenocarcinoma (CK7+ / CK20−) and ruling out metastatic colonic carcinoma to the lung (CK7−/ CK20+)^6^. In addition, CDX2, a highly sensitive and specific marker of adenocarcinoma of intestinal origin, was negative (**Figure 5**). Thyroid transcription factor-1 (TTF1) biomarker was positive, (**Figure 6**) distinguishing primary (TTF1+) from metastatic lung neoplasms (usually TTF1−).

## DISCUSSION

Although the two most frequent causes of death worldwide, cardiovascular disease and cancer, are often present together, malignant neoplasms leading to fatal cardiovascular events via vascular spread are extremely rare. Nevertheless, cancer and cardiovascular disease share many risk factors, such as tobacco use, poor nutrition and lack of physical activity. Also, chronic inflammation and oxidative stress play central roles in the pathophysiology of both^7^.

In an observational study, Sturgeon et al. revealed that one in ten cancer patients do not die from malignancy, but due to associated cardiovascular conditions, cancer patients have an on average 2–6 times higher mortality risk due to cardiovascular disease than the general population^8^.

In this exceptional case, the immediate cause of death was acute myocardial infarction of the anterior wall. Although the coronary arteries had no significant stenosis and he did not have risk factors for cardiovascular diseases, he had multiple recurrent infarctions. Malignant cells were detected in the coronary arteries, potentially due to tumor embolism via pulmonary veins or from aortic metastases. Therefore, the findings suggested coronary embolism from neoplastic cells.

Coronary embolism is the underlying cause of 3% of acute coronary syndromes. However, it is often not considered in the differential diagnosis of acute coronary syndromes9. Previously published papers reported only a few cases of myocardial infarction due to coronary artery tumor embolism, which was diagnosed from coronary artery aspirate10. The major source of cancer embolism to the coronary artery is the lung, apparently via the pulmonary veins, and the diagnosis is often made post-mortem^9-11^.

It has been reported that the incidence of coronary embolization in patients with myxomas is only 0.06%. Acute myocardial infarction due to lung carcinoma embolization to a coronary artery is even more exceptional^12^.

Secondary aortic manifestation of neoplastic disease is very rare. Similar to cases involving primary aortic tumors, arterial tumor embolization from secondary aortic involvement of neoplastic disease is very likely to be the cause of myocardial infarction in our case. Our patient had diffuse infiltration of the aortic wall by neoplastic cells that were located primarily in the vasa vasorum.

Many clinical manifestations may accompany aortic malignant neoplasms, regardless of whether the tumor is primary or metastatic. These include but are not limited to embolic events to different organs, aortic aneurysm and dissection.

Ikebe and colleagues described a case of arterial tumor embolization from aortic metastasis in a 67 year-old man with cholangiocarcinoma, with descending aortic wall thickening visualized by positron emission tomography-computed tomography. After 6 months, he had symptoms of mesenteric ischemia. Angiography with thromboaspiration was performed and histopathological analysis of the embolus demonstrated tumor emboli from cholangiocarcinoma metastasis^2^.

Aortic malignancies affect the structure of the medial layer of the aorta, and primary aortic tumors have previously been associated with aortic dissection^13-15^. Only few cases of aortic dissection caused by secondary tumors involving the aorta have been reported. Ugurlu et al. present a case of typical ascending aortic dissection associated with metastatic carcinoma originating from the lungs. This metastatic infiltration of the vasa vasorum of the aorta by neoplastic cells may have caused aortic dissection by altering the media^3^. Another case revealed at autopsy an intramural hematoma of the aorta, as well as systemic metastases of adrenocortical carcinoma with invasion into the aortic wall and formation of a pseudo-lumen accompanied by disruption of the vasa vasorum^4^.

In the early stages, tumor aneurysms are very similar to atherosclerotic aneurysms on computed tomography scan, often leading to a delay in diagnosis. The atypical localization of the aneurysm, low cardiovascular risk factors, rapidly evolving aneurysms and enhanced tissues around the aorta are suspicious findings for aortic metastasis. PET scan fluorodeoxyglucose uptake around the aneurysm, although not specific, can contribute to the diagnosis^5^.

## CONCLUSION

Cardiovascular events induced by malignant neoplasms are rare and can present with various clinical manifestations. Imaging techniques, such as PET-CT, could improve early diagnosis.

In patients with cardiovascular events, such as myocardial infarction or aortic dissection without known risk factors for cardiovascular disease, neoplastic disease should be considered as a differential diagnosis. This case illustrates the value of autopsy in unexpected deaths, as neither the underlying disease nor the immediate cause of death was suspected ante-mortem.

## KEY POINT

◊ Exceptionally, malignant neoplasms can lead to cardiovascular complications and death, via hemangiosis carcinomatosa with secondary infiltration of perivascular connective tissu
